# Developmental Dyslexia and Dysgraphia: What can We Learn from the One About the Other?

**DOI:** 10.3389/fpsyg.2015.02045

**Published:** 2016-01-26

**Authors:** Diana Döhla, Stefan Heim

**Affiliations:** ^1^Department of Psychiatry, Psychotherapy and Psychosomatics, Medical Faculty, RWTH AachenAachen, Germany; ^2^Institute of Neuroscience and Medicine, Research Centre Jülich Jülich, Germany

**Keywords:** developmental dyslexia, dysgraphia, German, DSM-V, ICD-10, comorbidity

## Abstract

Up to 17% of German school children suffer from reading and writing disabilities. Unlike developmental dyslexia, only few studies have addressed dysgraphia. Presenting a comprehensive overview of the current state of the art in developmental dyslexia and dysgraphia, this paper aims to determine how far existing knowledge about the causes of developmental dyslexia also apply to developmental dysgraphia. To promote understanding of developmental dysgraphia, the paper discusses relevant aspects such as predictors, causes and comorbidities, models of acquisition as well as existing deficit models. A comparison of definitions in the DSM-V and ICD-10 complemented by an overview of the most recent German guideline ought to give the reader deeper insight into this topic. The current issue of growing up bilingually and the connection between reading and writing deficits are also discussed. In conclusion, this paper presents a critical survey of theoretical and practical implications for the diagnostics and treatment of developmental dysgraphia.

## Introduction

Developmental dysgraphia is a disorder characterized by difficulties in the acquisition of writing skills, with writing performance below that expected based on children’s class level. It is closely related to developmental dyslexia, a disorder in the acquisition of reading skills, and like developmental dysgraphia, despite adequate visus, schooling, and other cognitive abilities. The prevalence for developmental writing disorders is about 7–15% among school-aged children, with boys being more affected than girls (Hawke et al., 2009; 2–3 times: Katusic et al., 2009). This percentage is similar to the incidence of developmental dyslexia, which is about 17% (Shaywitz and Shaywitz, 2005). The Diagnostic and Statistical Manual of Mental Disorders (DSM-V), the fifth edition of the classification by the American Psychiatric Association (2014) and an important diagnostic tool, estimates the prevalence of all learning disorders (including impairment in writing as well as in reading and/or mathematics) to be about 5–15% worldwide and the German S3 guideline names prevalence for reading and/or writing disorders of about 3–8%.

### Definition of Developmental Disabilities

#### Developmental Dyslexia and Dysgraphia

The clinical and scientific knowledge about developmental dyslexia has grown in the last years. Whereas developmental dyslexia has moved into the focus of research, the investigation of developmental dysgraphia has garnered less attention. Yet for both disorders, there are different classifications and definitions in the literature, making it difficult for the reader to gain insight into the actual characteristics and causes of these disorders and their relationship. In this regard, the current state of the art is summarized below.

#### A Comparison of DSM-V and ICD-10

In the following section, the DSM-V (American Psychiatric Association, 2014) is compared to the tenth edition of the International Statistical Classification of Diseases and Related Health Problems (ICD-10), a medical classification system established by the WHO (World Health Organization, 2015).

In the DSM-V, reading and writing disorders are found in the category of SLD. They are distinguished as “SLD with impairment in reading” [315.00 (F81.0)], “SLD with impairment in written expression” [315.2 (F81.81)], as well as “SLD with impairment in mathematics.” The first two subgroups are categorized further as:

• Specific learning disorder with impairment in reading can vary between problems in word reading accuracy, reading rate, or fluency and reading comprehension.
• Specific learning disorder with impairment in written expression is divided into problems with either spelling accuracy, grammar and punctuation accuracy and clarity or organization of written expression.

The subgroup “SLD with impairment in mathematics (dyscalculia)”^1^, is beyond the scope of this paper and, thus, is not discussed in detail here.

The ICD-10 also indicates the category of “developmental disorders of academic skills.” In contrast to the DSM-V, it only differentiates between impairment in reading and writing (F81.0) and isolated impairment in writing (F81.1). A single impairment in reading is not categorized. Furthermore, the ICD-10 names a “disorder in mathematics” (F81.2) and a combined disorder of academic skills that includes a “disorder in mathematic skills, reading and writing.” (F81.3). Finally, F81.3 as “other developmental disorder of academic skills including developmental expressive writing disorder” is classified. Unlike the ICD-10, the DSM-V specifies reading and writing disorders by current severity (mild-moderate-severe).

In the DSM-V, the trend is not to regard the discrepancy between IQ and reading ability any longer because of children who do not fit into the discrepancy criterion but who have the same problems with underlying deficits (Pennington and Bishop, 2009). Yet the definition of a developmental disorder in language or reading still demands an IQ greater than or equal to 70. The DSM-V cites some predictors such as avoiding language games, and speech and language difficulties at pre-school age. However, Snowling and Hulme (2012) criticize that the strong link between language and reading impairments should be described more precisely. Moreover, DSM-V and ICD-10 both do not make any recommendations about which tests should be used to best diagnose a reading or writing deficit. Likewise, they do not recommend any therapy methods.

It must be mentioned that DSM-V came out just recently, whereas DSM-IV and ICD-10 were closely related. It will be most interesting to learn the modifications introduced to the ICD-11 which is presently in preparation.

The following part gives a comprehensive overview of the current state of research, referring to possible comorbidities and causes of dyslexia and dysgraphia. As mentioned above, there is much more research for developmental dyslexia than for dysgraphia. Therefore, the question arises if knowledge about developmental dyslexia is also valid for developmental dysgraphia. To answer this, we need to focus on the differences between the process of reading and the process of writing.

## How Do Reading and Writing Disorders Differ?

In the literature, there are reports of a high correlation between word reading and writing performance (between *r* = 0.68 und *r* = 0.86, Ehri, 2000). The two skills have much in common: acquisition is similar with respect to the developmental phases. While knowledge about the alphabetic system plays an important role, the connection between the use of the semantic system does too. These facts lead to the conclusion that the underlying abilities necessary for reading and writing are likely similar, if not the same. But looking more closely, writing seems to be more demanding than reading. Five reasons for this theory are summarized in Winkes (2014) and described in the following part using the example of the German language:

(1) Phoneme-to-grapheme-correspondence is much more complex than GPC. Whereas there is usually only one possibility to read a word (GPC) in German, in writing (PGC) there are many different ways to realize phonemes.
(2) The second reason states that “full cues vs. partial cues” is closely associated with the first reason and refers to an incomplete or non-existing orthographic representation in the lexicon. It is easier to correctly identify a word for reading than to write a word correctly. With the help of the phonological reading route, a word can be read out correctly. In contrast, we can write an unknown word phonetically accurately, but there is a high risk not to write it orthographically correctly.
(3) Recall is known as a higher function than recognition. During reading, the visual representation of words only needs to be recognized. Writing is described as more complex. The orthographic representation has to be retrieved from the mental lexicon completely and independently.
(4) Poor readers benefit from the context. Constructions of sentences or texts limit the choice of words whereas linguistic setting obviously is not helpful while writing.
(5) Winkes (2014) names the effect of training. During life we spend much more time reading than writing. Because of the motoric process, writing takes longer than reading, described as a rapid and highly automatized process.

Winkes (2014) concludes that reading and writing are similar but not symmetric processes, and he confirms the metaphor of the “two sides of the coin- theory” introduced by Ehri (2000). Yet does this mean that developmental dyslexia and dysgraphia are two distinct disorders?

## Comorbidities and Predictors of Developmental Dyslexia and Dysgraphia

Assuming that the cognitive and motor requirements for writing differ in some important aspects from those for reading, we will now focus on the existing information about comorbidities and predictors of developmental dyslexia. Moreover, we will discuss the transferability of this knowledge to developmental dysgraphia.

Many studies have highlighted the connection between phonological processing difficulties and reading and writing disabilities. Phonological processing consists of three functions: PA, phonological working memory, and phonological recoding in lexical access (Ramus and Szenkovits, 2008). These three functions work separately but are correlated.

### Phonological Processing

#### Phonological Awareness

It is the ability to work with phonological structure of words like recognizing, segmenting, synthesizing, and manipulating phonemes, syllables and onsets and rhymes. The ability of grapheme-to-phoneme conversion, which is necessary for reading, and the ability of phoneme-to-grapheme conversion, necessary for writing, is part of the PA. Pennington et al. (2012) states that PA is the best single predictor for dyslexia. Written expressions represent the phonological structure of spoken language. As explained above, PGC is much more complex than GPC. To write orthographically correct, i.e., identifying phonemes is indispensable; many orthographic rules can be derived by segmenting words into syllables, for instance. Thus, PA is an important ability not only for reading but also for writing (Moll et al., 2009, 2012).

#### Phonological Working Memory

It consists of a passive phonological buffer and an active process of phonological rehearsal which are both combined in the phonological loop (Baddeley, 2003). Based on these two modules, information can be kept in the buffer for a short time. Using the phonological loop and active rehearsal processes, the individual can retain information longer. Consequently, this system keeps phonological representation during the working process complete and in the correct order (Glück, 2000). Repeating an increasing amount of syllables or words in the same order can test working memory (Menghini et al., 2011). To demonstrate the link between reading and working memory, Menghini et al. (2011) compared a group of dyslexics with normal readers. They found out that dyslexic children not only scored worse in phonological components but also in visual-object and visual-spatial working memory than the group of age-matched normal readers.

Even for reading comprehension in early and later elementary school children, phonological working memory plays an important role, because it keeps information active while the persons read phrases or sentences (Seigneuric and Ehrlich, 2005).

These results from the reading domain are at least partly transferable to writing. During writing, working memory has to keep information upon building phoneme-to-grapheme conversion, synthesizing and segmenting phonemes to words. Furthermore working memory is important for building up orthographical representation and for linking these representations with phonological and semantic information (Winkes, 2014).

#### Phonological Recoding in Lexical Access

It means the activation of the correct phonological code and meaning in the mental lexicon to a visual stimulus. To test the speed of access from long-term memory to phonological code, rapid automatized naming (RAN) tasks are used (Norton and Wolf, 2012). These tasks consist of naming letters, numbers, colors, and objects as quickly as possible. The connection between deficits in phonological recoding in lexical access was shown in different studies (Mayer, 2008; Pennington and Bishop, 2009; Pennington et al., 2012). Diverse authors describe poor results in RAN and PA as early predictors of reading ability (Norton and Wolf, 2012; van Ermingen-Marbach et al., 2013, 2014; Pape-Neumann et al., 2015). Wolf and Bowers (1999) and Wolf et al. (2000, 2002) describe an effect which combines deficits in PA and NS. In their study, they emphasize four subtypes of readers: (1) children with average skills in PA and NS, (2) children who show poor results in PA but average skills in NS, and (3) children who show poor results in NS but average skills in PA, and (4) children who exhibit deficits in PA as well as in NS. The outcome of the fourth group is called the double-deficit-hypothesis.

Yet do low scores in RAN tasks predict a deficit in writing skills as well? Winkes (2014) compared German speaking six-graders with isolated writing deficits to those with isolated reading deficits. Children who are below average only in writing showed good results in RAN tasks but significant worse results in PA. It seems that results in RAN tasks show different results for German readers and writers.

In recent years, however, the focus on phonological abilities as relevant for reading and writing disorders has somewhat expanded to now include also visual and auditory processing abilities, visual and auditory attention, or automatization (Nicolson et al., 2001; Valdois et al., 2003; Bosse et al., 2007; Nicolson and Fawcett, 2007, 2011; Reid et al., 2007; Pennington and Bishop, 2009).

### Auditory Processing

Ramus (2003) summarizes that many studies have already confirmed the link between auditory processing and dyslexia. In his review, he discusses the connection between auditory processing and phonological deficit. He concluded that both a phonological deficit and a deficit in auditory processing can appear independently in dyslexic children. It needs to be kept in mind that a severe auditory impairment nonetheless can have a negative influence on phonological skills and therefore affect reading and writing (Ramus, 2003; Ramus et al., 2003). Steinbrink et al. (2014) tested German dyslexic children with respect to phonological and auditory processing skills (temporal and spectral). Their study also demonstrated that substandard skills in auditory processing may explain deficits in phonological processing and might therefore, in some cases, be regarded as causal for phonological deficits. Importantly, Steinbrink et al. (2014) reported differential deficit profiles in the dyslexic children: some with only temporal processing difficulties, some with only spectral deficits, and one with an isolated phonological deficit. In most cases, however, if one of those three dimensions was affected, the deficit co-occurred with at least one other, or even all deficits. Christmann et al. (2015) found similar results for adult dyslexics with respect to temporal, spectral, or spectrotemporal deficits.

### Visual-Phonological Deficits

Mirror writing and kinetic reversals (e.g., /was/ and /saw/, /b/ instead of /d/) were long considered as a main symptom of dyslexia with repetitions in reading and writing as well as the omission of sounds and misspelled letters being handled as secondary symptoms (Orton, 1925). Orton (1937) established the theory that the two sides of the brain code spatial information oppositely. Some mistakes, such as confused consonants, e.g., f/v or g/c could not longer only be regarded as a visual deficit but rather seen as a phonological problem. This phonological aspect in connection with dyslexia led to discussions about multi- and unicausal models (for a comprehensive summary see Corballis and Beale, 1993; Lachmann, 2002) still being discussed today (see the following sections).

### Visual-Magnocellular Processing

Until now, many studies have confirmed the further hypothesis in connection with the visual aspect that dyslexia can be based on a deficit in the magnocellular system (cf. Tholen et al., 2011). This theory is based on an impairment of the brain’s magnocellular system which supports processing of rapidly moving visual stimuli and, thus, is responsible for saccadic eye movements. An impaired magnocellular system causes blurred visual representation of letters, for example. As a result, letters are more difficulty to distinguish. Dyslexics therefore have difficulties with detecting fast movements and misperceive seemingly moving letters (Stein, 2001).

The connection between dysgraphia and an impaired magnocellular system has not been proven until now. But the problem of not clearly seeing letters in their correct orders, as a consequence of a magnocellular deficit (Stein, 2014), can cause individuals to write letters in a wrong order. This error can be transferred to dysgraphic children.

### Attention and Attention Deficits

Facoetti et al. (2003) describe the relation between reading disorders and auditory and visual deficits in the orienting spatial attention. Based on this fact, Facoetti suggests a distortion of development of phonological and orthographic representation as an indirect consequence of the deficit in spatial attention that, in turn, impairs a child’s learning to read. Since phonological and orthographic representations are also accessed during writing, this implies that attention has an impact on the acquisition of writing skills as well. Indirect evidence for this notion comes from the study by Rosenblum et al. (2004). These authors assessed handwriting of dysgraphic children. They summarize research and describe the problem of dysgraphics having not fully automatized letter production. Therefore, dysgraphics have an increased demand on their memory and attention while writing. Consequently, higher-level cognitive processes are constrained. As a further limitation, these authors describe the fact that children may forget plans held in memory before they are able to write them down on paper because of slow handwriting. This deficit may lead to serious consequences for students’ academic process, their emotional well-being and their social function. Rosenblum et al. (2004) confirm the characteristics of dysgraphic writing such as consistently lower quality of individual spatial writing features. This includes inconsistent letter size, acute turns in letters, uneven and unsteady writing as well as sudden changes in size and direction of letter writing.

Finally, Adi-Japha et al. (2007) investigated the connection between clinically relevant attention deficits in patients with ADHD and reading and writing skills. In summary, they determined that clinically relevant attention problems cause problems in writing. They suggested that the deficits in writing do not necessarily have underlying linguistic problems but an impaired graphemic buffer and impairments in kinematic motor production. Compared to children without ADHD, their test subjects with ADHD made a higher amount of spelling errors in the morphological categories (function words, free morphemes, and derivational words). Yet the reading abilities of the ADHD-test subjects were similar to those of the comparison group. Children with ADHD scored worse in letter insertion, substitutions, omissions, and transpositions (graphemic buffer tasks). They changed similarly shaped letters. However, the results in fluency were similar for both groups.

Furthermore, tests on motor kinematic production showed poor time utilization which means that children with ADHD take longer for writing. The test subjects displayed inconsistent and disproportionate handwriting so that their handwriting was difficult to read. A high amount of corrections and writing with high levels of pressure on the pen for subjects with ADHD was pointed out as well (Adi-Japha et al., 2007). These findings refer back again to the Rosenblum et al. (2004) data discussed above.

### Automatization Deficit

**FIGURE 1:**
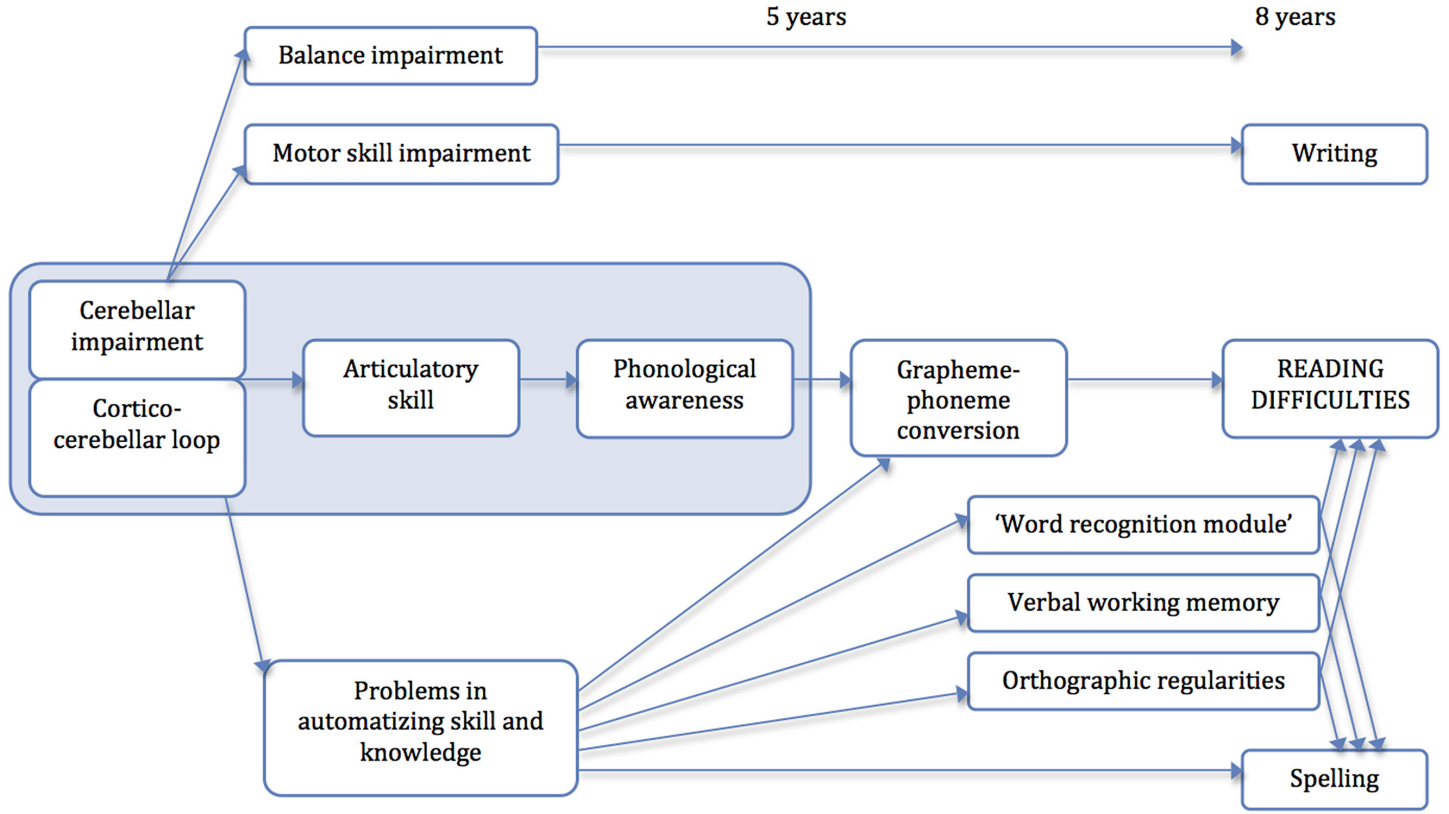
**A causal chain of the cerebellar-deficit-hypothesis (cf. Nicolson and Fawcett, 2011)**.

Proficient readers master GPC without problems because of their normally developed basic articulatory and auditory skills. It is assumed that the cerebellum supports the automatization of these basic abilities. Consequently, learning and automatization of, e.g., grapheme-to-phoneme correspondence as well as other PA tasks is complicated with a dysfunctional cerebellum. The cerebellum processes motor skills and coordination as well as linguistic and cognitive skills (Ito, 2008). Nicolson et al. (2001) state that an impairment of motor skills resulting from an impaired cerebellum adversely affects writing skills. That means that a dysfunctional cerebellum can lead to reading as well as writing disorders (Nicolson et al., 2001). Nicolson and Fawcett (2011) developed a possible causal chain of the influence of cerebellar impairment on reading and writing skills (see **Figure 1**). Writing problems, thus, ought to be caused by motor skill difficulties. Reading problems arise from problems in phonology, based on deficient articulatory skills that stem from cerebellar impairment. In addition, spelling problems result from a deficit in skill automatization. The authors point out that the activated regions of the cerebellum differ for each of the three routes. Hence, a dyslexic person may be impaired in writing (motor skill) and/or reading and/or spelling.

Nevertheless, the current state of research showed diverse outcomes with no significantly worse results between dyslexics and controls with respect to automatization tasks (Ramus et al., 2003; Heim et al., 2008 tested with dyslexic adults). The group of test subjects with the worst scores in automatization tasks (rhythm imitation paradigm), tested by Heim et al. (2008), also displayed poor skills regarding other cognitive functions. Therefore, a larger multiple cognitive deficit was concluded. By contrast, earlier studies demonstrated a connection between automatization and dyslexia (Fawcett et al., 1996; Tiffin-Richards et al., 2001). Nicolson and Fawcett (2007) proposed a neural-system approach. They stated that dyslexics have an intact declarative but impaired procedural learning system with activity in diverse brain areas such as the prefrontal language system, basal ganglia and cerebellar structures (for a detailed overview of the neural activation, see Nicolson and Fawcett, 2007). The authors base their theory on the fact that dyslexic children have problems to automatize knowledge so that explicit attentive control is no longer necessary. They developed a three-stage automaticity-deficit-framework of skill learning to explain potential additional motor skills (also shown in **Figure 1**) in dyslexic children: in an initial declarative stage, a person learns what to do; in an intermediate stage the person learns how to do it; and in a final autonomous stage, the skill becomes fluent and automatic. In a response-blending study (Nicolson and Fawcett, 2000) the authors found out that dyslexic children have problems in the initial stage and take longer for intermediate proceduralization. The authors declare that these findings are consistent with the known role of the cerebellum regarding skill automatization or point to a communication problem (Nicolson and Fawcett, 2007).

These aforementioned authors describe five stages of learning: fast learning (minutes), slow learning (hours), consolidation (overnight), automatization (hundreds of trials), and retention (weeks). The authors worked out that dyslexics can have problems in one or more of these stages, e.g., in fast-learning stage or automatization stage because of not being able to activate the cerebellum (Nicolson and Fawcett, 2007). Consequently, the authors summarize that because of an impairment of procedural learning caused by an impaired cerebellum, children with dyslexia and dysgraphia have problems to automatize skills (Nicolson and Fawcett, 2011).

### Specific Language Impairment as a Predictor of Dyslexia/Dysgraphia?

Specific Speech Disorder, LI and developmental dyslexia are multifactorial disorders with respect to genetic, environmental and cognitive etiology; all include deficits in phonological processing (Pennington and Bishop, 2009). Pennington and Bishop (2009) found comorbidity of LI and later developmental dyslexia but a lower risk for SSD and later dyslexia. The combination of SSD and LI leads to the highest risk of reading disorder. No grammatical limitations were found in children with dyslexia in combination with LI (Bishop and Snowling, 2004). These authors created a two-dimensional model that demonstrates the relationship between dyslexia and LI on the basis of phonological deficits. This model also allows for possible additional disordered domains of oral non-phonological language skills. This confirms the importance of diagnosing the different underlying disorders as explained above.

With respect to writing skill deficits, oral language difficulties seem to be a predictor as well. Children with LI perform worse in tasks like writing names, letters, and spelling words in comparison to a control group (Puranik and Lonigan, 2012).

### Familial Disposition

Olson et al. (1989) determined that phonological coding and segmental language skills, such as rhyming and phoneme segmentation, are highly inheritable. By contrast, orthographic coding was not significantly inheritable. They assume that a deficit in orthographic coding can be a result of environmental factors. Parent education and parent reading history seem to have no effect on reading skills (Pennington et al., 2012). Thompson et al. (2015) found that the family risk of dyslexia is a strong predictor of literacy outcome. Schulte-Körne et al. (2006) found inheritability of word reading (50–60%), as well as of spelling (50–70%).

Given this wide range of abilities other than phonological skills, which seem to be relevant for the successful acquisition of reading skills, Heim et al. (2008) investigated the potential existence of cognitive subtypes in school-aged dyslexic children, and they observed three different clusters:

Dyslexic children in Cluster 1 were characterized by phonological, visual, and auditory deficits. The children categorized in Cluster 2 performed worse only in PA whereas those in Cluster 3 performed worse in terms of visuospatial attention. These results confirm the heterogeneity of dyslexia and the need to diagnose individually, considering the possibility of deficits in these domains which might influence poor reading skills.

As indicated in Heim et al. (2008), Heim and Grande (2012) and Pennington et al. (2012), the causes of dyslexia and also the comorbidities may vary substantially depending on the individual child’s profile. In most cases, dyslexia seems to accompany multiple deficits or may represent a single deficit with variation, e.g., in PA, language skills, or NS.

Even so, the phenomenon of dysgraphia requires further investigation. As discussed above, it can be safely assumed that diverse underlying disorders must be regarded for diagnosing developmental dysgraphia as well. First evidence for this hypothesis was given with respect to ADHD and hand-eye coordination. The risk factor of LI and PA seems to be similar for developmental dyslexia and dysgraphia, whereas the difference between skills in RAN-tasks underscores the hypothesis that developmental dyslexia and dysgraphia are not symmetric disorders (Winkes, 2014).

Even if a detailed description of intervention of developmental dyslexia and dysgraphia^2^ goes beyond the scope of this article, it is noteworthy that these new insights about comorbidities and predictors ought to have an important influence on future research on intervention.

To obtain an overview of diagnostic procedures, we will consider the German S3 Guideline “Diagnosis and intervention of children and adolescents with reading and writing disorders” (AWMF, 2015) in the following section. This guideline, which targets an interdisciplinary audience (physicians, therapists, pedagogs), gives a survey of current research regarding diagnosis of developmental reading and writing disorders and corresponding intervention methods.

## Diagnostic Criteria of Reading and Writing Performance

The authors of the German S3 guideline recommend the expert to regard a discrepancy of at least 1.5 SD (standard deviation) between the outcome of an intelligence test (in comparison to standard norms like the IQ or other age- or grade-related norms) and reading and/or writing performance (Hoskyn and Swanson, 2000; Stuebing et al., 2002; AWMF, 2015). The performance in particular tests of those different learning domains should be 1 SD below the arithmetic mean. The authors also suggest a diagnosis of impaired vision as well as peripheral hearing disorder as exclusion criteria.

Furthermore, the authors recommend different tests to diagnose reading and/or writing impairment based on criteria such as objectivity, retest-reliability, validity, description of theoretical principles of material and a representative norming sample of *n* ≥ 250. The same criteria were used to evaluate tests for underlying disorders (e.g., deficit in PA). Yet for the German language, no tests were found that fulfill these criteria (AWMF, 2015).

In concluding, it can be said that, in addition to knowledge about discrepancy criteria and so on, information is given about different recommendable tests for reading and writing. This can be a helpful support for the practitioner. The German guideline clearly worked out that no adequate materials for (young) adults as well as terminally appropriate tests for underlying disorders exist.

## Deficit Models of Reading and Writing

In the current literature many different models, especially of subtyping dyslexia, have been published. As already mentioned, an individual diagnosis of reading, writing and especially of underlying skills is inevitable. Yet only one underlying deficit, e.g., impaired PA, is allowed. Since there are many cases where more than one function is impaired, it is challenging to describe dyslexia with a theoretical model. In the literature the debate concerns choosing the right type of model – single vs. multiple or hybrid deficit models (Pennington et al., 2012).

Connectionist models show the importance of phonology and semantics for speech, language, and reading development. A problem in phonology or semantics can affect one or more of the developmental skills (Pennington and Bishop, 2009).

These authors criticize the problem of models only dealing with single deficits instead of multiple deficits on comorbidities. Pennington et al. (2012) investigated two central questions: “Whether a single deficit is necessary and sufficient to cause dyslexia or not” and “Whether a deficit in PA is necessary to cause dyslexia or not.” Therefore they compared cognitive single- versus multiple deficit models and one hybrid model (comparison can be found in Pennington et al. (2012)). They found that these models partially encompass one another. PA is included in some models and is seen as a prerequisite for the diagnosis of dyslexia. Other models, however, allow another deficit instead of PA, or deficits in PA as well as in NS (double-deficit-hypothesis; Wolf and Bowers, 1999; Wolf et al., 2000, 2002; Pennington et al., 2012). The authors conclude that one single phonological model probably is not adequate because of the high amount of predictive reading deficits (Pennington et al., 2012). A model should allow a single underlying deficit as well as multiple impaired skills. Finally their study revealed that a hybrid model that encompasses all four possibilities of cognitive single- and multiple deficit models (**Table 1**) best depicts the causes of dyslexia. For the sake of comparison, it should be noted that nearly all dyslexics tested by Pennington et al. (2012) had at least one marginal (11–25th percentile) cognitive deficit (PA, language skills (both semantic and syntactic/morphological) and processing speed and/or NS).

**Table 1:** Four cognitive models of dyslexia (Pennington et al., 2012).

		**Single deficit?**	
		
		**Yes**	**No**
**PA Deficit Necessary?**	**Yes**	I: Single phonological deficit	III: Phonological core, multiple deficit
	**No**	II: Single deficit subtypes	IV: Multiple deficit


The question arises whether developmental dysgraphia can be depicted with such a hybrid model as well. As discussed above, developmental dysgraphia also seems to show diverse underlying deficits. A single deficit in PA is possible (Moll et al., 2009, 2012). Analogously to the Pennington et al. (2012) model, a single deficit, for example, in visual attention is plausible as well (even though not envisioned, e.g., in the Rosenblum et al., 2004 study). Yet a model that allows multiple cognitive deficits such as impaired PA, working memory as well as an underlying LI is theoretically feasible, too. Therefore, it can be speculated that the findings explained above can be transferred to developmental dysgraphia: a hybrid model that allows a single or multiple cognitive deficits might be a good foundation to explain developmental dysgraphia. To this end, studies of individual profiles of dysgraphia (e.g., Ramus et al., 2003; Reid et al., 2007; Heim et al., 2008 for dyslexia) are urgently needed.

In line with this argumentation, an earlier investigation by Ziegler et al. (2007) underlines the importance of diagnosing in a multiple way. Analyzing dyslexics by testing the different representational levels (letter level, phoneme system, orthographic and phonological lexicon) of the DRC; Coltheart et al., 2001) showed a diversity of triple, dual, single and no further deficits on dyslexics. Ziegler et al. (2007) conclude that it is also problematic to use a strict model to diagnose dyslexics because of their individual characteristics.

## Dual-Route Models of Reading and Writing

**FIGURE 2:**
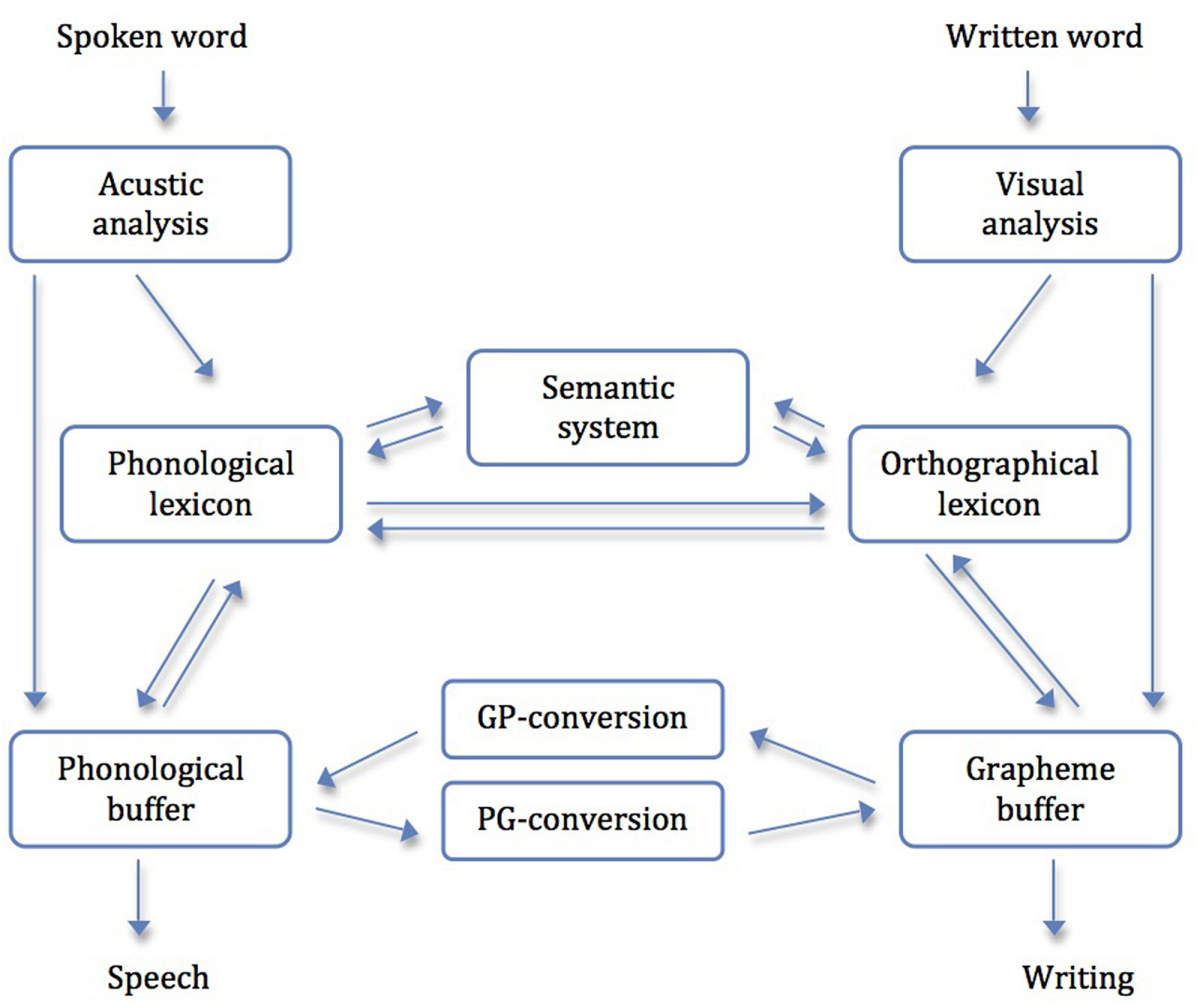
**The dual route model of reading and writing (cf. Winkes, 2014)**.

Because of the high importance for reading and writing performance and for better understanding, the dual-route-model (**Figure 2**) will be explained briefly in the following part. The starting point for the development of this model was the ‘logogen model’ by Morton (1969). In the first version, the model only explained the reading of single words; since 1980 writing was added. Since then it had been further developed many times, inter alios by Coltheart (2005, 2006) as the DRC. Reading and writing are built up as mirror-inverted processes. Moreover, the box-and-arrow model explains different possibilities of reading and writing by using different routes and components. With the non-lexical route, reading and writing of pseudowords by using PGC and GPC is possible. When one uses the lexical route, already known words are recalled from the orthographical/phonological lexicon. Words acquire their meaning when one activates the semantic system. With the help of this component, the reader and writer can differentiate between homophones like “right” and “write.”

The transferability of those models to diagnose developmental reading and writing disabilities was shown by different authors (e.g., Barry, 1994; Costard, 2007). However, one should bear in mind that the theory of the models is based on research with patients with acquired disorders. Assets and drawbacks are discussed later on.

## Models of Reading and Writing Acquisition

The developmental framework of Frith (1985) serves as the basis for later developmental models. Written language development is subdivided in different phases: logographic, alphabetic, and orthographic strategy. Other authors, e.g., Scheerer-Neumann (2008), further elaborated the stage model of writing and reading. The following table gives an overview of the main phases of reading and writing acquisition of a normally developing child in a language with rather transparent orthography (**Table 2**).

**Table 2:** Reading and writing acquisition.

	Time	Writing
**(1) Logographic strategy**	Before starting school	• Initial knowledge about writing
		• First words can be written without phonemic knowledge, e.g., proper name
**(2) Alphabetic strategy**	First and second grade (6–8 years)	• Learning PGCs
		• Learning to write phonetically accurately
		• Learning words by learning orthographic anomalies by heart
		• Writing pseudowords by using PGC
**(3) Orthographic strategy**	Third and fourth grade (8–10 years)	• Writing difficult words by applying orthographic rules
		• Learning explicit orthographic rules as guidelines for spelling unknown words
	Fifth and sixth grade	• Children have acquired all orthographic special features of words

	**Time**	**Reading**

**(1) Logographic strategy**	Before starting school and first grade	• Only familiar words can be analyzed using essential features, e.g., according to the proper name or brand names like McDonald’s with the help of the characteristic “golden arches” M
**(2) Alphabetic strategy**	First grade	• Learning PGCs to read unknown words and pseudowords
**(3) Orthographic/lexical strategy**	Second grade	• Reading words in larger units, e.g., in morphemes instead of letter-to-sound
		• Most words are represented in the lexicon and can be accessed as a whole


## Reading and Writing Across Orthographies

The regularity of a writing system, i.e., its orthographic transparency, affects how easily children learn to read (Snowling and Hulme, 2012). Thus, transparent alphabetic orthographies such as German, Italian, Czech, and Finnish are easier and faster to learn than English or French, for instance. Less is known about the alphasyllabaries (e.g., Korean) or logographic languages (e.g., Chinese, Japanese kanji) and how easily children learn to read and write (Snowling and Hulme, 2012).

The psycholinguistic grain size theory (Goswami, 2002; Ziegler and Goswami, 2005) describes the connection between PA and reading acquisition across languages. Depending on the particular language, the orthography varies in its phonological representation and its phonological consistency. The authors assume that the size of the units, used for reading according to the non-lexical route, varies between children and adults, depending on the orthographic consistency of the different languages. Therefore, there are developmental differences across languages. In orthographic consistent languages, a faster development of phoneme-level skills can be observed (e.g., Finnish) than in languages like English.

It may be useful to differentiate between alphabetic languages (e.g., Finnish, Italian German, or English) and logographic languages (e.g., Chinese). The first type varies in the consistency of letter-sound correspondence from a high consistency (e.g., Finnish) to a low consistency (e.g., English). Landerl et al. (1997) found out that children learning a language with a high consistency of letter-sound correspondence have fewer deficits in reading than those learning to read less consistent languages whereas skill of reading fluency is independent of language. PA remains the main predictor of reading across languages and plays a greater role in highly consistent orthographies (Ziegler et al., 2010). Perhaps contrary to expectations, PA plays an important role in learning to read Chinese as well. Less surprisingly and in contrast to alphabetic languages, morphological and syllabic awareness are highly important for learning to read Chinese (McBride-Chang et al., 2005).

As for reading acquisition, PA also serves as a core component for spelling development. Furthermore, letter knowledge plays an important role for writing acquisition across alphabetic languages (Caravolas, 2004). Caravolas and Bruck (1993) compared English and Czech children with respect to their phonologically accurate pseudoword spellings in first grade. They discovered that children who learn orthographically consistent languages like Czech show faster spelling development than children who learn the less orthographically consistent language of English. Furthermore, children learning more orthographically consistent languages seem to learn not only the basic spelling skills like pseudoword spelling more easily but also more advanced spelling skills (cf. Caravolas, 2004).

## Mono- vs. Bilingual Acquisition of Reading and Writing

Bialystok et al. (2005) determined that children who grow up bilingually have a general advantage in reading acquisition. Children learning two alphabetic languages especially benefit while learning to read. The authors state that bilingual children who learn alphabetic languages like Hebrew or Spanish as a second language and English as the first language performed better in PA tasks than monolingual and bilingual children who spoke English and Chinese (as a non-alphabetic-language). The link between the advantage gained in PA tasks for bilingual children learning two alphabetic languages implies that bilingual children may somehow benefit in writing acquisition as well –depending on the similarity or distinctness of the individual languages a child grows up with. To the best of our knowledge, no data are currently available which shed light onto this question.

## Conclusion

We now return to the initial question about to which extent do developmental dyslexia and developmental dysgraphia share common features. In this paper, we have discussed the following core facts.

• Writing is considered to be a much more complex process than reading.
• Both disorders – developmental dyslexia and developmental dysgraphia – can exist together as well as separately.
• Both learning disabilities have in common that they have diverse comorbidities and predictors within and outside the language domain. PA plays an important role for dyslexia and dysgraphia as well. Moreover, it can be assumed that further predictors and comorbidities such as working memory, auditory processing, visual attention, and LI play an important role in reading and writing as well. Future research on this topic is needed to prove this assumption.

However, the relevance of those comorbidities and predictors seems somewhat different for dyslexia and dysgraphia. For instance, even though PA is important for reading and writing skills, if we take a deeper look at the reading and writing process, we can see crucial differences between the two. If we explain this by using the alphabetic strategy/writing or reading pseudowords, as an example, we can say that synthesizing phonemes to words plays the most important role for reading, whereas for writing, segmenting words in phonemes is more important. Moreover, whereas on the one hand performance in RAN-tasks is only predictive for developmental dyslexia, on the other hand hand-eye coordination is relevant only for writing performance. Finally, the roles of other domains such as attention or working memory clearly require further research.

Altogether, the present state of the art seems to suggest that developmental dyslexia and developmental dysgraphia have a rather broad common underlying basis, in particular, in the domain of phonological processing, and a high amount of co-occurrence. It also becomes clear that still too little is known about the relationship between reading and writing, or dyslexia and dysgraphia with respect to other cognitive, sensory, or motor domains.

## Outlook

Developmental dyslexia has often been a focal point of research in the past years. Peterson and Pennington (2015, p. 288) pointedly conclude, “The scientific study of difficulties in written expression […] is at a much earlier stage of development.” Thus, it is essential to acquire more information especially about developmental dysgraphia. Two lines of research open up here. The one is the extension of the far more detailed reading/dyslexia models to writing/dysgraphia. It may prove helpful to include the notion of individual profiles, or even subtypes, of dyslexia to the domain of dysgraphia. The second is related to this topic and focusses on applying these novel models and concepts for individualized diagnosis and therapy. The German guideline (AWMF, 2015) already gives a helpful overview. Yet more robust evidence, in particular from RCTs (randomized controlled trials), are needed to demonstrate the relevance and external validity of these novel concepts by reducing the number of non- or low-responders to dysgraphia intervention and by increasing the overall success rate. The present overview has aimed to promote this endeavor by indicating what aspects of developmental dysgraphia we may learn from research on developmental dyslexia.

## Conflict of Interest Statement

The authors declare that the research was conducted in the absence of any commercial or financial relationships that could be construed as a potential conflict of interest.

## Footnotes

1 Dyscalculia is known as a specific deficit in the acquisition of mathematics skills (Landerl et al., 2009). The DSM-V differentiates between difficulties mastering number sense, number facts or calculation and difficulties with mathematical reasoning. Various studies have shown the comorbidity of dyscalculia and dyslexia/dysgraphia. Landerl and Moll (2010) found a four- to five-times increased rate for children already suffering from one learning disability. Wilson et al. (2015) found a comorbidity rate of around 40% for dyslexia and dyscalculia. Nevertheless, the degree of similarity between the underlying problems in dyslexia and dyscalculia remains partly unknown. Landerl et al. (2009) compared dyslexic with dyscalculic children. Their findings demonstrated that dyslexia and dyscalculia have different cognitive profiles – an inadequate number module for dyscalculic children and poor phonological results for the dyslexics. Children affected by dyslexia and dyscalculia showed abnormalities in both areas. Wilson et al. (2015), in contrast to Landerl et al. (2009), found out that the two disorders have “bridge symptoms” namely speed of lexical access (tested as naming speed) as a core symptom and working memory as a second symptom. Dyscalculia therefore seems to be an independent disorder that often coexists in dysgraphic children.

2 Effectiveness of intervention methods for improving reading and writing skills can be derived, in particular, from meta-analysis of randomized controlled trials (Galuschka et al., 2014; AWMF, 2015). The recent literature gives evidence that intervention should be adjusted to individual needs. This includes training of reading and/or writing skills as well as training of underlying skills depending on the individual situation. Exercises to improve phonological awareness skills including grapheme-to-phoneme and phoneme-to-grapheme correspondence as well as exercises of segmenting words into phonemes, syllables, morphemes, and onset-rhyme and synthesizing phonemes to words should be selected. In addition, systematic exercises with sentences and texts are recommended. With the help of the individual composition of these exercises, improvement in reading fluency, and writing phonetically accurately can be expected (Galuschka et al., 2014; AWMF, 2015). The authors of the German S3 guideline recommend the trainer not to use only one training program, because the effectiveness could not be proved yet. They state that intervention, at best, should combine different existing programs.

## Abbreviations

ADHD: attention deficit hyperactivity disorder
DRC: dual route cascaded model of reading aloud
DSM-V: diagnostic and statistical manual of mental disorders, the fifth edition
GPC: grapheme-to-phoneme-correspondence
ICD-10: International Statistical classification of diseases and related health problems-tenth revision
LI: language impairment
NS: naming speed
PA: phonological awareness
PGC: phoneme-to-grapheme-correspondence
RAN: rapid-automatized naming
RCT: randomized controlled trail
SD: standard deviation
SLD: specific learning disorder
SSD: specific speech disorder
WHO: World Health Organization
